# PSO-Based Dynamic RSU Role Assignment Framework for Scalable and Reliable Content Delivery in VANETs

**DOI:** 10.3390/s26051555

**Published:** 2026-03-02

**Authors:** Yongje Shin, Hyunseok Choi, Youngju Nam, Euisin Lee

**Affiliations:** 1 School of Information and Communication Engineering, Chungbuk National University, Cheongju 28644, Republic of Korea; yjshin@cbnu.ac.kr; 2 Department of Computer Engineering, Seowon University, Cheongju 28674, Republic of Korea; hschoi@seowon.ac.kr; 3 School of Software, Kunsan National University, Kunsan 54150, Republic of Korea; imnyj@kunsan.ac.kr

**Keywords:** vehicular ad-hoc networks, cluster RSU, particle swarm optimization, dynamic content replication, multi-user content delivery

## Abstract

Vehicular Ad-hoc Networks (VANETs) must sustain heterogeneous real-time content services, yet static roadside-unit (RSU) roles lead to congestion, coverage voids, and inefficient content delivery under bursty, concurrent demand. To address this issue, we propose a PSO-Based dynamic RSU role assignment framework named PDRA that dynamically adapts roles, coverage, and replication of RSU to current network conditions. A telemetry-based suitability estimator aggregates location, link stability, resource availability, traffic load, and content sensitivity at each RSU and feeds a Particle Swarm Optimization routine that assigns RSUs to Leader/Helper/Inactive roles while enforcing spatial separation between Leaders. An adaptive sectoring mechanism then resizes each cluster RSU’s communication scope—contracting under overload to protect local latency and expanding during slack to assist neighbors—thereby suppressing void areas and balancing service density. On top of the physical layer of RSUs, Leader RSUs cooperatively form a virtual Replication Layer that maintains global visibility of load and content locality to steer requests and replicate popular data near demand, reducing backhaul reliance. Finally, a load- and energy-aware reconfiguration policy orchestrates staged assist/offload, selective Helper activation, PSO-based Leader reassignment, and sleep scheduling for underutilized RSUs, preserving resilience and sustainability. NS-3 urban scenarios corroborate that PDRA improves packet delivery, lowers end-to-end delay, reduces backhaul traffic, and increases fairness over strong baselines. By jointly optimizing role assignment, coverage control, and replication, PDRA offers a scalable and robust solution for VANET content delivery under dynamic, multi-user conditions.

## 1. Introduction

Vehicular Ad-hoc Networks (VANETs) [1] have emerged as a cornerstone of Intelligent Transportation Systems (ITS) [2], providing critical infrastructure to support seamless real-time communications among vehicles (V2V) [3] and between vehicles and roadside infrastructure (V2I) [4]. These networks have enabled numerous advanced applications, including autonomous driving, real-time traffic management, emergency alerts, infotainment, and multimedia content delivery [5,6,7]. As vehicles increasingly integrate connectivity and autonomous functionalities, the role of VANETs continues to grow in importance, driving significant attention from researchers and industries.

Despite the considerable progress in VANET technologies, the highly dynamic nature of vehicular environments presents several inherent challenges. High mobility of vehicles results in rapidly changing network topologies and frequent disruptions in connectivity, making stable communication difficult to maintain. Moreover, the highly variable density of vehicles, especially in urban areas, creates unpredictable traffic patterns, fluctuating loads on the communication infrastructure, and localized traffic congestion. These dynamics pose considerable challenges in maintaining efficient and reliable data transmission [8].

Road Side Units (RSUs) have become essential components in VANET systems, primarily tasked with relaying information, distributing content, and supporting communication between vehicles and the broader network infrastructure. Traditionally, RSUs are installed at fixed locations with predefined coverage areas, which limits their flexibility to adapt dynamically to changing traffic patterns and user demands. Consequently, traditional static RSU deployments frequently encounter overloaded conditions, communication delays, and coverage gaps, especially during peak usage or in highly dynamic traffic scenarios.

The increasing demand for high-quality multimedia services, such as video streaming, live navigation, and real-time traffic monitoring, further exacerbates these limitations. Multimedia services require high bandwidth, low latency, and robust connections, requirements difficult to satisfy under conventional RSU setups [9]. When multiple users concurrently request content, static RSU deployments often fail to distribute network resources effectively, leading to degraded service quality and reduced user satisfaction.

In recent years, several approaches have been proposed to address these issues, including adaptive RSU management, dynamic caching, content replication strategies, and advanced clustering techniques. These approaches primarily attempt to dynamically adjust RSU resources and operational strategies in response to changing network conditions [10]. However, many existing solutions rely heavily on predefined, rigid strategies or simplified adaptation mechanisms, which lack the necessary responsiveness and adaptability required for rapidly changing vehicular environments.

Particle Swarm Optimization (PSO) [11], a metaheuristic algorithm inspired by the collective behavior of social organisms such as bird flocks and fish schools, has gained prominence due to its ability to efficiently solve complex optimization problems. PSO has notable advantages for real-time applications, including simplicity, quick convergence, and minimal computational complexity. These characteristics make PSO particularly attractive for optimizing dynamic scenarios prevalent in VANETs, where decisions regarding RSU deployment, positioning, and resource allocation must be made rapidly based on continuously changing conditions.

Recognizing these potentials, this paper proposes a novel PSO-based Dynamic RSU Role Assignment Framework incorporating a dynamic replication mechanism specifically designed to improve multi-user content delivery efficiency in VANET environments. Our proposed framework introduces a dynamic replication-aware topology management layer to support real-time dynamic sector resizing and adaptive resource allocation, significantly enhancing system responsiveness and scalability.

Within the proposed framework, each cluster RSU functions as a dynamically adaptive content provider, strategically adjusting its coverage and resources in real-time based on vehicular density, localized demand patterns, and content replication needs. By prioritizing vehicles located within its immediate transmission range and adapting its service area dynamically, each cluster RSU ensures optimal resource utilization and minimizes void-area regions with insufficient coverage due to ineffective RSU placement.

### Contributions

The primary contributions of this paper are summarized as follows:

1. **Dynamic PSO-Based Cluster RSU Selection:** We propose a novel application of Particle Swarm Optimization (PSO) for dynamically selecting optimal Cluster RSU locations and roles based on real-time vehicular density and traffic patterns, significantly improving responsiveness and network adaptability.
2. **Replication-Aware Topology Management:** Our proposed framework incorporates a replication-aware dynamic sector resizing mechanism, enabling efficient management of concurrent multi-user content requests and enhancing load balancing across the network.
3. **Adaptive Coverage Adjustment:** The developed Cluster RSU protocol dynamically adjusts RSU coverage areas based on localized vehicle distribution and user demands, effectively reducing void areas and ensuring consistent content delivery quality.
4. **Real-Time Resource Allocation:** By dynamically allocating RSU resources and adapting transmission strategies, our proposed framework significantly enhances multimedia content delivery performance, reducing latency and improving overall performance for users.
5. **Comprehensive Performance Evaluation:** We conduct extensive simulations using realistic vehicular mobility models and network scenarios, demonstrating substantial performance improvements in packet delivery ratio, latency, throughput, and system overhead compared to traditional static RSU solutions.
6. **Scalable and Robust Solution:** Our methodology provides a scalable and robust solution that can seamlessly adapt to varying network conditions and future VANET expansions, laying a strong foundation for subsequent research and real-world deployment.

This article is organized into several sections, each addressing a core component of the proposed framework for VANET optimization. Section 2 presents a comprehensive review of related work in five key domains: RSU deployment strategies, content caching and replication, swarm intelligence applications, load balancing mechanisms, and dynamic topology adaptation techniques. Section 3 introduces the proposed PSO-based cluster RSU framework, detailing its multi-phase optimization structure and replication-aware meshing mechanism. Section 4 outlines the simulation environment and performance evaluation metrics used to assess the effectiveness of the proposed framework under realistic vehicular mobility and mixed-content request conditions. Section 5 discusses the results, analyzing the impact of the protocol on service responsiveness, resource utilization, and scalability. Finally, Section 6 concludes the paper by summarizing the contributions and outlining directions for future research in infrastructure-aware, adaptive vehicular networking. Unlike existing approaches that focus on single-objective optimizations—such as load balancing (AALB), caching (HRL-PC), or routing (PSUV)—without modifying the physical infrastructure settings, PDRA proposes a joint optimization framework. Specifically, PDRA is the first to simultaneously adapt the RSU role, coverage radius, and content replication layer to dynamically handle spatiotemporal demand fluctuations. A detailed qualitative comparison between PDRA and these state-of-the-art baselines is provided in Section 5.1.

## 2. Related Work

While various efforts have been made to address the challenges of dynamic vehicular environments, many existing studies focus primarily on the initial deployment or static optimization of RSUs, aiming to maximize coverage or minimize deployment costs. These approaches, although effective for basic infrastructure planning, are often insufficient to handle real-time fluctuations in traffic density, user demand, and network load that characterize modern VANET scenarios. In response to these limitations, researchers have explored techniques such as content-aware caching, load-balanced replication, and cluster-based role assignment to improve service availability and resource utilization. More recently, swarm intelligence algorithms have been employed to enhance decision-making processes in vehicular networks, including RSU selection, clustering, and adaptive communication strategies.

However, a common limitation across many of these works is the assumption of either fixed RSU roles or coarse-grained adaptation mechanisms that lack responsiveness to rapid, localized changes in the network. Furthermore, existing role assignment strategies often fail to consider the complex interplay between concurrent multi-user content requests, real-time load status, and inter-RSU cooperation. To address these gaps, our proposed framework integrates a PSO-driven cluster RSU selection algorithm with a dynamic, replication-aware meshing mechanism. This design enables each RSU to not only assume adaptive roles based on resource suitability and traffic conditions, but also dynamically adjust its coverage and collaborate with neighboring cluster RSUs to balance load and eliminate void areas.

In the following sections, we review related literature in five key domains that form the foundation of our work: (1) RSU Deployment and Management in VANETs, (2) content caching and replication strategies for vehicular environments, (3) swarm intelligence and PSO-based optimization approaches, (4) load balancing and void area mitigation techniques, and (5) dynamic topology adaptation and real-time content request handling.

### 2.1. RSU Deployment and Management in VANETs

RSU deployment has been widely studied as a multi-objective planning problem that balances spatial coverage, latency, and cost under realistic traffic conditions. While this literature has advanced beyond simple heuristics to incorporate demand heterogeneity and robustness constraints, most approaches concentrate on the initial layout and treat RSU roles and coverage regions as static once installed, limiting adaptability to short-term surges and localized hot spots.

For example, the work by Yu et al. [12] proposed a traffic-demand-driven placement strategy that mines spatial variation in vehicle flows to identify high-impact intersections and corridors. By prioritizing persistent demand regions, the scheme improves connectivity and delay at a given budget. However, the design assumes fixed post-deployment operation, offering no mechanism for runtime role coordination or elastic coverage when neighboring cells experience sudden load spikes.

In a complementary direction, Wang et al. [13] proposed an improved multi-objective QPSO (MOQPSO) formulation that treats RSU siting as a maximum-coverage-with-time-threshold problem. Their method co-optimizes the number, spacing, and service radius of RSUs and achieves superior Pareto fronts over common metaheuristics. Yet, once the layout is chosen, operational parameters remain static—there is no inter-RSU cooperation layer, replication plan, or sector resizing to accommodate bursty multi-user demand.

To enhance resilience, Zeng and He [14] proposed a bi-level, failure-aware deployment model for mixed AV/HV traffic, maximizing total connectivity/coverage (upper level) while capturing stochastic user equilibrium in routing (lower level). An improved binary PSO yields robust layouts under RSU failure probabilities and communication-radius constraints. Still, the contribution addresses where to place RSUs rather than how deployed RSUs should coordinate roles, replicate content, or adapt coverage at runtime.

Expanding the planning space, Jain et al. [15] proposed MOURD, a multi-objective UAV-assisted RSU deployment strategy that augments terrestrial infrastructure by dispatching aerial relays to sparse regions. This improves coverage without dense ground roll-outs but remains a planning-time augmentation; it does not provide an in-operation mechanism for role reconfiguration among terrestrial RSUs or replication-aware coordination across cells.

In contrast to prior deployment-centric approaches—such as demand-driven siting, multi-objective (Q)PSO layouts, failure-aware placement under mixed traffic, and UAV-assisted augmentation—which concentrate on planning-time optimization and consequently fix roles and cell boundaries, the proposed framework performs run-time orchestration of deployed RSUs. Using PSO, it continuously reconfigures Leader/Helper roles based on instantaneous load, link quality, and content locality.

Through a replication-aware meshing layer, it proactively disseminates popular objects and redistributes request backlogs across neighboring cluster RSUs; and via elastic sector reconfiguration, it absorbs excess demand into underutilized cells to suppress void regions and localized overload. This infrastructure-level coordination directly addresses bursty, concurrent content requests, reducing tail latency, increasing sustained throughput/PDR during surges, and improving resilience to partial failures—thereby translating planning-time optimality into sustained, in-operation performance gains.

### 2.2. Content Caching and Replication Techniques

Content caching and replication are essential techniques for reducing access latency and alleviating bandwidth bottlenecks in vehicular networks, especially under high-demand multimedia and real-time service conditions. While previous studies have made significant strides in predictive and cooperative caching, most focus narrowly on local cache decision-making without broader inter-RSU coordination or dynamic role-based adaptation.

For example, the work by Ahmed et al. [16] proposed a fog-assisted caching strategy for 5G vehicular networks that leverages a Gale–Shapley stable matching algorithm to associate RSUs with nearby fog nodes. This pairing is based on multiple weighted criteria, such as content popularity, expected vehicle dwell time, and wireless link quality. The system aims to assign popular contents to fog nodes best suited to serve them, thereby reducing response latency and alleviating backhaul congestion. However, this approach assumes that each RSU operates in a relatively isolated caching domain and does not account for dynamic interactions or content replication between RSUs, limiting its ability to rebalance service loads when sudden demand surges occur in adjacent regions.

In a more mobility-sensitive context, Zhang et al. [17] proposed a mobility-aware vehicular caching scheme for content-centric networks in which moving vehicles act as cache carriers. They model vehicle–user encounters via a two-dimensional Markov process and formulate an online caching decision as an energy efficiency optimization using nonlinear fractional programming and Lyapunov techniques, yielding gains in energy efficiency, cache hit ratio, cache utilization, and overall system gain over non-vehicular or offline baselines. Nevertheless, control remains localized to vehicle-level caching (and associated access), with no cross-RSU coordinated cache management or explicit online replication/migration in response to sudden density shifts or multi-user contention.

To improve proactive caching decisions, recent studies have incorporated machine learning and reinforcement learning techniques. Wang et al. [18] proposed a cooperative caching strategy that predicts vehicular content requests and applies Q-learning to optimize cache placement and provider selection. By mining historical request patterns and mobility-aware features, the framework groups vehicles with similar demand profiles and pre-positions content at likely hotspots, improving cache-hit ratio and reducing miss probability and delay. However, it assumes fixed RSU roles and geographic boundaries, offering no adaptive role reassignment, inter-RSU coordination, or sector resizing in response to transient overloads.

A more advanced method is proposed in Wang et al. [19], which applies Hierarchical Reinforcement Learning (HRL) to manage caching decisions across a vehicle-edge-cloud collaborative architecture. The system divides caching tasks between multiple layers, with higher-level agents deciding when to cache, and lower-level agents selecting the optimal caching location. This structure enables flexible and intelligent cache placement across the edge-cloud continuum. Still, the model treats RSUs as fixed entities and lacks an embedded mechanism for peer-to-peer coordination, dynamic role migration, or replication-aware load balancing, all of which are critical in high-concurrency scenarios with unpredictable demand.

Similarly, Lin et al. [20] proposed a coalition game-based distributed cooperative caching (CGD3C) scheme for vehicular edge networks, in which RSUs self-organize into coalitions to jointly cache and deliver large multimedia objects. Coalition formation is utility-driven—balancing caching gains against coordination and resource costs—which reduces duplication and improves cache-hit efficiency. However, the coalition configuration is not explicitly coupled to real-time telemetry (e.g., instantaneous RSU load, bursty request surges, or coverage overlap), and the design lacks an adaptive role-reallocation mechanism to react to emerging void areas or congestion along coalition boundaries.

Lastly, Wang et al. [21] proposed a multi-armed bandit (MAB) learning model to proactively cache content by predicting future RSU-user associations. The system estimates which RSUs a vehicle is likely to connect to in the near future, based on contextual information such as vehicle trajectory, average speed, and network connectivity history. While the method achieves better early-stage content availability and reduces cold-start latency, it fundamentally focuses on RSU-level caching decisions. It does not incorporate inter-RSU coordination, cache migration, or replication protocols that could better address multi-user concurrent requests or spatial content demand imbalance.

In contrast to these approaches, our proposed framework combines PSO-based real-time cluster RSU role selection with a replication-aware dynamic meshing system that allows RSUs to flexibly adjust their service areas and redistribute cached content collaboratively. This design directly addresses several limitations seen in the existing literature—such as fixed-role RSU structures, lack of replication across sectors, and the inability to respond to localized load surges—ultimately enabling a more scalable, balanced, and responsive caching system in high-mobility vehicular environments.

### 2.3. Swarm Intelligence and PSO Applications in Network Optimization

Swarm intelligence techniques, particularly Particle Swarm Optimization (PSO), have attracted increasing attention in vehicular network research due to their ability to solve complex optimization problems with minimal overhead and rapid convergence. PSO has been widely applied to enhance routing efficiency, resource allocation, and offloading decisions within VANETs. However, the majority of these approaches focus on either vehicle-centric or local RSU-level optimization, often lacking the structural coordination mechanisms necessary to support scalable and adaptive infrastructure roles.

For instance, Desai et al. [22] proposed a PSO-based data dissemination protocol to enhance routing in VANETs by selecting the optimal path from multiple candidates generated through Three-Multipath Routing (TMR). The system evaluates each path using delay-based fitness values and employs PSO to determine the most efficient route for message delivery. While this approach significantly reduces end-to-end delay and improves delivery reliability, it is fundamentally constrained to vehicle-level routing optimization. It lacks any consideration for infrastructure-level control such as RSU collaboration, adaptive sector formation, or content-aware role assignment, rendering it sufficient in managing multi-user traffic or volatile regional load distribution.

Tang et al. [23] proposed a utility-optimized resource allocation scheme for RSU-empowered vehicular cloudlets, where PSO is used to dynamically allocate computing resources among vehicles based on demand and latency sensitivity. The system demonstrates superior performance over GA and greedy algorithms in terms of responsiveness and throughput. However, the optimization occurs in isolation at each RSU, without any inter-RSU synchronization or role-aware coordination. The framework does not support real-time adaptation of RSU roles, sector reconfiguration, or replication-aware data handling, thus limiting its scalability and robustness in dynamic, high-density traffic scenarios.

A more advanced use of PSO appears in Shu et al. [24], who proposed a Quantum PSO (QPSO) approach to solve the joint offloading problem in MEC-enabled vehicular networks. The method uses adaptive expansion and quantum-inspired operators to efficiently assign computational tasks either to edge servers or nearby vehicles, aiming to minimize energy consumption and processing delay. Although algorithmically sophisticated, this model is confined to computational logistics and does not extend its optimization to RSU functionality or network-wide service continuity. It neglects the cooperative management of RSU resources, dynamic coverage tuning, and replication mechanisms essential for handling concurrent content demands.

Shin et al. [25] proposed a PSO-driven content delivery protocol that integrates UAVs with VANET infrastructure to address the void area problem. PSO is used to classify candidate UAVs based on their proximity and ability to maintain connectivity, enabling reliable content dissemination in sparsely covered regions. Although this protocol effectively mitigates service gaps through aerial assistance, it is highly dependent on the UAV infrastructure and does not establish any collaborative framework among ground RSUs. It lacks the capacity to assign RSU roles dynamically, adaptively replicate content, or manage load fluctuations across RSU sectors, all of which are central to our proposed meshing framework.

In summary, while the aforementioned studies demonstrate the versatility of PSO and its variants in vehicular networks—ranging from routing optimization to resource allocation and offloading—most are limited to isolated optimization objectives and lack a holistic infrastructure coordination framework. They generally overlook the need for dynamic RSU role assignment, replication-aware cooperation, and adaptive sector-wide coverage control, which are critical for ensuring consistent service quality in environments with fluctuating vehicular density and concurrent user demands. In contrast, our proposed framework integrates PSO-based Leader RSU and Helper RSU selection with a replication-aware meshing mechanism, enabling real-time load balancing, void area mitigation, and scalable content delivery through coordinated RSU interactions.

### 2.4. Load Balancing and Void Area Mitigation in Vehicular Networks

Vehicular networks often face unbalanced RSU utilization and void areas due to dynamic traffic conditions and uneven vehicle distribution. These challenges lead to degraded service quality, especially for latency-sensitive applications. Recent research has proposed various solutions such as load-aware routing, anomaly detection, and mobile RSU deployment to alleviate congestion and improve coverage. However, most approaches rely on static architectures or local decision-making, lacking real-time role reconfiguration and collaborative RSU coordination.

For instance, Sahoo et al.’s Application-Aware Load Balancing (AALB) algorithm [26] focuses on optimizing RSU workloads in vehicular networks by using virtual machine (VM) migration. The system classifies vehicular service applications based on latency and criticality requirements, and proactively shifts VM workloads from overloaded RSUs to lightly loaded neighbors. The core innovation lies in balancing application responsiveness with migration cost, using resource-level load indicators and deadline constraints. However, the approach assumes a largely static RSU infrastructure and does not incorporate real-time vehicle density changes or cooperative role delegation among RSUs. It also lacks a replication framework, limiting its capacity to mitigate service voids or adapt to simultaneous content requests from densely populated regions.

Ni et al. [27] proposed a hybrid RSU management system under the Cybertwin-IoV paradigm, wherein fixed RSUs (sRSUs) and mobile RSUs (mRSUs) are jointly managed through centralized utility-based scheduling. This system predicts temporal and spatial traffic distribution and dispatches mRSUs to cover emerging high-load regions or underserved areas. The hybrid structure is effective in reducing access latency and extending coverage flexibility, particularly in fluctuating urban traffic conditions. Nonetheless, the design focuses on RSU positioning and does not provide mechanisms for in situ role transitions (e.g., Leader to Helper), dynamic meshing, or content replication across RSUs. As a result, it cannot adaptively reassign service loads or mitigate void areas under bursty and concurrent user demand.

Song et al. [28] proposed STALB (Spatio-Temporal Autonomous Load Balancing), which shifts the focus from infrastructure-centric control to message-level congestion avoidance. STALB leverages spatio-temporal vehicle movement patterns to make anticipatory packet-forwarding decisions, thereby minimizing delay and alleviating congestion hotspots. The protocol adapts in real time to vehicle trajectories, intersection density, and route volatility. While STALB excels in vehicular message scheduling and real-time path control, it is confined to network-layer forwarding optimization and does not incorporate RSU-based cooperation, coverage control, or infrastructure-level resource balancing. In particular, it does not dynamically reassign RSU responsibilities or replicate content to neighboring zones to absorb traffic surges.

Dai et al. [29] proposed a comprehensive framework for joint load balancing and offloading for Vehicular Edge Computing (VEC) systems. By jointly optimizing edge server selection and computation task offloading through a mixed-integer nonlinear programming model, the system improves user fairness, load distribution, and latency minimization. The optimization accounts for bandwidth constraints, task deadlines, and server capacities. However, the framework is server-centric and assumes fixed offloading nodes without addressing RSU collaboration, dynamic role assignment, or physical service region adaptation. It does not handle scenarios where RSUs must dynamically reshape their coverage or delegate roles to maintain balanced service distribution.

Finally, Laanaoui et al. [30] proposed a Lambda-based data-processing architecture to analyze real-time traffic patterns and vehicle density. Leveraging machine-learning-based anomaly detection, it flags atypical traffic spikes and dynamically adjusts routing to mitigate congestion and improve flow. The framework enhances urban traffic management through multi-layer data fusion and predictive re-routing. However, despite its strength in traffic-level intelligence, it does not integrate RSU-level control, provide mechanisms for content replication, or address RSU-level failure, overload, or coordination. Consequently, it remains insufficient for managing physical-infrastructure constraints in dense or mission-critical environments.

In summary, while existing approaches provide valuable contributions to load balancing and congestion mitigation in vehicular networks, they often focus on isolated layers such as message routing, computation offloading, or static RSU control. Most lack dynamic RSU role assignment, inter-RSU collaboration, and replication-aware adaptability. In contrast, our proposed framework unifies these capabilities through PSO-based cluster RSU selection and real-time meshing, enabling infrastructure-level responsiveness to load shifts, void area formation, and concurrent user requests in a scalable and coordinated manner.

### 2.5. Dynamic Topology Adaptation and Real-Time User Request Handling

As vehicular networks grow increasingly dynamic and service-intensive, maintaining stable topology and responding to fluctuating user requests in real time has become a critical challenge. Rapid changes in vehicle density, mobility patterns, and content demands often render static routing and fixed clustering schemes insufficient. In response, recent studies have proposed various mobility-aware and traffic-adaptive protocols to support dynamic topology formation and service continuity. However, most existing approaches focus on vehicle-level clustering or predictive routing and lack infrastructure-level coordination, role-based RSU adaptation, or replication-aware handling of concurrent multi-user requests.

El Alaoui et al. [31] proposed MADCR, a mobility-aware dynamic clustering-based routing protocol for the Internet of Vehicles. Their scheme employs the Mayfly Optimization Algorithm (MOA) to select cluster heads (CHs) based on Euclidean distance and vehicle mobility features. This enables stable and efficient cluster formation in highly dynamic traffic environments, thereby improving data delivery and reducing end-to-end latency. However, the architecture focuses solely on vehicular-level coordination and lacks any RSU-side intelligence. Without infrastructure-level load balancing, dynamic RSU role assignment, or content replication mechanisms, the protocol is unable to effectively handle concurrent user requests or adapt to rapidly changing network conditions beyond local clusters.

Goudarzi et al. [32] proposed TACRP, a Traffic-Aware Clustering-Based Routing Protocol designed to improve routing efficiency through centralized clustering using a Traffic Management Unit (TMU). Vehicles are grouped based on mobility similarity (speed and direction), and clusters communicate with RSUs via selected cluster heads. The scheme effectively reduces packet loss and improves routing stability under high traffic density. Nonetheless, TACRP assumes fixed RSU roles and offers no strategy for dynamic coverage adjustment or inter-RSU coordination. In the absence of a replication-aware topology adaptation mechanism, the protocol cannot address service voids or load surges during sudden traffic shifts or concurrent content delivery requests.

Dhakad et al. [33] proposed a dynamic clustering-based, risk-aware congestion control strategy utilizing DBSCAN and K-means to organize vehicles into adaptive clusters. The system detects congestion and forms “virtual safety regions” to manage communication range and channel contention. While this enhances safety message reliability and reduces broadcast overhead, the approach is entirely focused on vehicle-to-vehicle communication and lacks integration with the RSU infrastructure. As such, it cannot support infrastructure-assisted request handling, role-based RSU adaptation, or coordinated sector-wide load redistribution—key features required for scalable, real-time service delivery.

Ragab et al. [34] propose a mobility-aware vehicular cloud formation mechanism for edge computing environments. Their system forms on-the-fly vehicular clouds by dynamically selecting nodes based on location, connectivity, and mobility metrics. This enables temporary collaboration among vehicles to offload tasks from edge servers. Although the approach supports real-time mobility adaptation and opportunistic computation, it operates without RSU collaboration and is limited to local cloud formation. It lacks the capability for long-term service continuity, dynamic role delegation, or cross-cell resource sharing, making it unsuitable for handling persistent user demand or ensuring consistent Quality of Experience (QoE) across heterogeneous VANET infrastructures.

While the reviewed approaches exhibit commendable progress in enabling mobility-aware clustering and adaptive communication under dynamic vehicular conditions, they predominantly operate at the node level and lack explicit support for infrastructure-driven coordination. The absence of mechanisms for RSU role reconfiguration, replication-aware service distribution, and cross-sector adaptability limits their effectiveness in managing concurrent user demands and maintaining topological stability. In contrast, the proposed framework advances beyond these limitations by integrating PSO-based cluster RSU selection with a dynamic meshing framework, thereby facilitating scalable, resilient, and infrastructure-aware adaptation to real-time vehicular and service-level dynamics.

### 2.6. Recent Advances in Distributed and Energy-Aware Coordination

Recent studies have extensively explored distributed coordination and energy-aware mechanisms in vehicular edge computing. Wang et al. [35] proposed an efficient task scheduling scheme explicitly considering the dynamic states of edge servers to improve load balancing and resource utilization in VEC environments. In the domain of intelligent content delivery, Zhang et al. [36] introduced a mobility-aware cooperative caching framework that utilizes Asynchronous Federated Learning (AFL) combined with Deep Reinforcement Learning (DRL) to adaptively optimize content placement under rapid topology changes. More recently, Schembra et al. [37] presented MANTRA, a fully distributed multi-agent framework based on Multi-Armed Bandits (MAB). This approach effectively optimizes offloading decisions to strike a balance between processing latency and energy consumption in roadside infrastructure.

While these works provide significant advancements in specific control domains (e.g., scheduling, caching, or offloading) through distributed or learning-based methods, our proposed PDRA framework distinguishes itself by focusing on the **joint optimization of infrastructure configurations**—specifically, the RSU role, coverage radius, and the replication layer—to proactively mitigate coverage voids and demand hotspots.

## 3. System Model and Preliminaries

### 3.1. VANET Topology and Node Framework

In the proposed framework, we model the vehicular ad-hoc network (VANET) as a dynamic and resource-aware graph G=(V,E,W), where *V* denotes the set of nodes, including both vehicles and Road Side Units (RSUs), *E* represents the set of wireless communication links among nodes, and *W* is the weight matrix that captures link quality and heterogeneous resource metrics.

Each node $v_{i}\in V$ periodically broadcasts its status vector, which includes the following:

- Geographical position and mobility state;
- Available computational resources: CPU, storage, and bandwidth;
- Current service demands or content requests (e.g., video streaming, sensor feeds);
- Communication reachability and link reliability to neighbor nodes.

The RSUs are semi-static infrastructure units that play a dual role in communication and edge computing. They are the primary candidates for being elected as Leader RSUs or Helper RSUs, based on their suitability determined by local context and global topology. Vehicles, on the other hand, act as dynamic consumers or relays within the network.

This graph-based representation provides the foundational structure for RSU scoring, clustering, and replication mechanisms introduced in subsequent sections. The system continuously updates *G* in real-time as node positions and network conditions evolve, enabling adaptive and decentralized optimization for service continuity and load balancing.

### 3.2. Particle Swarm Optimization (PSO) Fundamentals

Particle Swarm Optimization (PSO) is a population-based metaheuristic algorithm inspired by the social behavior of birds flocking or fish schooling. In the context of VANET optimization, PSO is employed to determine the optimal set of RSUs to be assigned as *Leader RSUs* and *Helper RSUs*, based on various resource and topology-aware fitness criteria.

Each particle in the swarm represents a potential configuration of RSU role assignments over the network. The position of a particle $x_{p}$ encodes a selection vector indicating which RSUs are selected and what their assigned roles are. The velocity $v_{p}$ determines how the configuration evolves over time. Each particle updates its state by considering its own best-known position $p_{\mathrm{best}}$ and the globally best-known solution $g_{\mathrm{best}}$ in the swarm:

(1) $$v_{p}\left(t+1\right)=\omega v_{p}\left(t\right)+c_{1}r_{1}\left(p_{\mathrm{best}}-x_{p}\left(t\right)\right)+c_{2}r_{2}\left(g_{\mathrm{best}}-x_{p}\left(t\right)\right)$$

(2) $$x_{p}\left(t+1\right)=x_{p}\left(t\right)+v_{p}\left(t+1\right).$$

where ω is the inertia weight, $c_{1}$ and $c_{2}$ are cognitive and social acceleration coefficients, and $r_{1},r_{2}∼U\left(0,1\right)$ are random values to ensure stochastic behavior. We evaluate each particle by the scalar fitness $F(x_{p})$ (see (11) and (12)): the first two terms sum the per-RSU scores $f_{i}$ and $f_{j}$ (defined in (7)–(9)) over selected Leaders and Helpers, while the penalty Ψ discourages overly redundant leader–leader placements and overly tight helper–leader clustering.

The fitness function $f(x_{p})$ for each particle is designed to balance the following objectives:

- Maximizing available RSU resources (CPU, bandwidth, storage);
- Minimizing traffic congestion and coverage overlap;
- Enhancing role diversity and spatial distribution of selected RSUs;
- Satisfying proximity constraints: $R\leq D_{\mathrm{leader}}\leq 2R$ to allow redundancy without excessive overlap.

This optimization process enables the system to dynamically and adaptively select RSU roles based on real-time traffic, node density, and content demands, ensuring load-balanced, resource-efficient operation across the network.

### 3.3. K-Means-Based Sector Partitioning and Replication Layer Design

To improve spatial scalability and enable localized optimization, the proposed framework partitions the entire network topology into logical geographic sectors using K-means clustering. Each sector represents a sub-region of the VANET where RSU role selection is performed independently to minimize interference and in balance.

Unlike traditional grid-based or density-based partitioning (e.g., DBSCAN), K-means clustering dynamically forms sectors based on the spatial distribution of vehicles and RSUs. Given a desired number of clusters *K*, the K-means algorithm iteratively assigns nodes to the nearest centroid and updates the centroids to minimize intra-cluster variance. This results in well-separated sectors with balanced node density.

Each RSU is uniquely assigned to one of the K clusters, forming the basis for sector-specific Particle Swarm Optimization. Within each sector, the PSO algorithm determines the most suitable Leader RSUs and Helper RSUs based on localized traffic load, connectivity, and available resources.

Once sector-level roles are assigned, the selected Leader RSUs across all sectors cooperate to form a global *Replication Layer*. This layer acts as a logical content delivery backbone, where Leader RSUs coordinate to distribute vehicle content requests and share cached data across cell boundaries. A centralized virtual controller oversees this layer to balance global load and reassign tasks under changing conditions such as resource depletion or RSU failures.

This hierarchical design—sector-level localized control and global replication coordination—ensures that the system adapts to both micro-level fluctuations (within sectors) and macro-level dynamics (across the network).

### 3.4. Traffic and Request Model

In vehicular ad-hoc networks (VANETs), traffic dynamics and service requests vary significantly with time, location, and vehicle density. To model these behaviors, we define a real-time traffic and request framework that captures the intensity and type of demands posed by moving vehicles across the network.

Each vehicle periodically generates service requests $R_{j}=\left\{l_{j},c_{j},t_{j}\right\}$, each of which is defined as follows:

- $l_{j}$: the current location of the vehicle;
- $c_{j}$: the content type requested (e.g., video streaming, sensor data, map updates);
- $t_{j}$: the timestamp of the request.

The RSUs in the network maintain per-cell request queues and aggregate traffic load metrics. For each RSU *i*, we define a real-time traffic load indicator $\rho_{i}$ as:

$$\rho_{i}=\frac{\lambda_{i}}{\mu_{i}}$$

where $\lambda_{i}$ is the arrival rate of requests and $\mu_{i}$ is the service rate based on available resources (CPU, bandwidth, and storage). The system employs two thresholds:

- $T_{\mathrm{assist}}$: If $\rho_{i}>T_{\mathrm{assist}}$, the RSU initiates load balancing via nearby Helper RSUs or adjacent cells.
- $T_{\mathrm{fail}}$: If $\rho_{i}>T_{\mathrm{fail}}$, emergency fallback mechanisms are triggered, including role reassignments via PSO.

In addition, the request model incorporates *content sensitivity*, where content types are prioritized based on their latency tolerance and resource demands. For example, real-time video streams receive higher weight in RSU scoring functions compared to non-real-time data such as file downloads or environmental sensing.

By integrating location-aware, time-sensitive, and content-prioritized traffic modeling, the system can dynamically assign roles and distribute load, ensuring responsive and efficient content delivery in a highly mobile and fluctuating VANET environment.

## 4. PSO-Based Dynamic RSU Role Assignment (PDRA) Framework

### 4.1. Overview of the PDRA Framework

**Figure 1:**
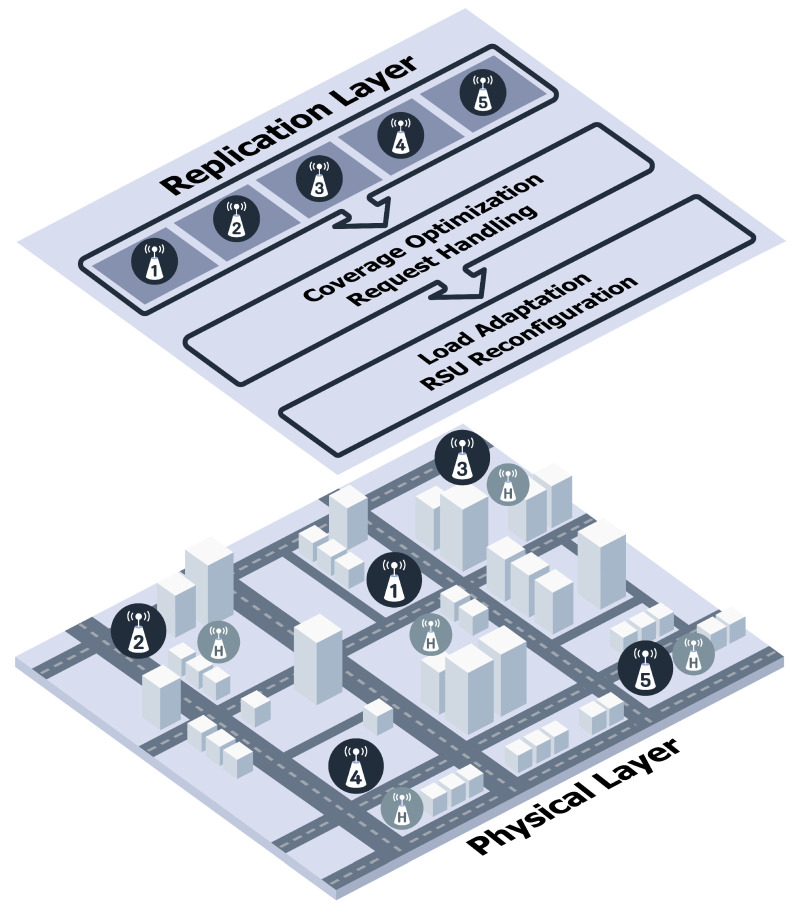
Operation across the Physical and Replication Layers: Physical—RSU1–RSU5 grouped; H nodes as Helpers. Replication—(1) coverage optimization (shape regions, reduce overlap); (2) request handling (dispatch by location and load); (3) load adaptation and RSU reconfiguration (assist/offload, Helper activation, role updates on threshold violations).

Algorithm 1 details the execution flow of the proposed framework, which addresses the limitations of static RSU deployments. The process operates in a periodic loop where RSU states are monitored; if the load exceeds a specific threshold ($T_{assist}$), the system triggers an adaptation phase to optimize RSU roles and radii using K-means and PSO, whereas a stability mode is maintained otherwise. This workflow integrates four cooperating modules: Real-time RSU Suitability Estimation, PSO-based cluster RSU Role Assignment with Adaptive Sectoring, Replication Layer Coordination and Request Routing, and Load Adaptation and Energy-aware RSU Reconfiguration. Figure 1 presents the operation across the Physical and Replication layers. From the Physical Layer, RSU1–RSU5 are logically grouped and elevated to the Replication Layer, while adjacent nodes labeled H act as Helpers. Within the Replication Layer, control proceeds in the following order: (1) *coverage optimization* to shape service regions and reduce overlap, (2) *request handling* to dispatch vehicle requests to the most suitable RSU group given location and instantaneous load, and (3) *load adaptation and RSU reconfiguration* to trigger assist/offload, activate Helpers, and update RSU roles when predefined thresholds are violated.
**Algorithm 1** Overall Workflow of the PDRA Framework1:**Input:** RSU states $S_{RSU}$, Vehicle demands $D_{veh}$, Thresholds $T_{assist},T_{fail}$2:**Output:** Optimal Roles, Radius $R_{i}$, Replication Map3:**Initialize:** Monitor RSU loads periodically ($\Delta T_{state}$)4:**while** System is Active **do**5:      Collect Entity Vectors $E_{i}$ from RSUs6:      **if** $\exists \;\mathrm{RSU}\;\mathrm{load}\;\rho_{i}>T_{assist}$ **then**7:            **Trigger Adaptation Phase:**8:            1. Partition Network into *K* sectors (K-means Clustering)9:            2. **Execute PSO Optimization** (in parallel for sectors):10:               - Minimize Cost Function $F_{cost}$ (Equation (9))11:               - Update Roles (Leader/Helper) and Radius $R_{i}$12:               - Update Replication Strategy13:           3. Disseminate Decisions to RSUs14:     **else**15:            Maintain current configuration (Stability Mode)16:     **end if**17:     Wait for next epoch ($\Delta T_{control}$)18:**end while**

#### MEC-Based Controller and Control-Plane Operation

PDRA employs a centralized virtual controller deployed on an edge MEC server interfacing with RSUs via backhaul. RSUs periodically report compact state summaries consistent with the entity vector (e.g., location, CPU, storage, available bandwidth, traffic load, residual energy/sleep state, and content-context indicators). Based on these summaries, the MEC controller disseminates updated decisions for RSU roles (Leader/Helper/Inactive), adaptive radius, and replication directives.

The control plane operates in an epoch-based loop: state reporting every $\Delta T_{\mathrm{state}}$ and PSO-based re-optimization with policy commitment every $\Delta T_{\mathrm{control}}$, with a minimum holding time $T_{\mathrm{hold}}$ to avoid oscillations. If the MEC controller becomes temporarily unreachable due to backhaul disruption, RSUs keep the last committed role/radius/replication plan for a bounded grace period (a few control epochs) and rely on local Leader–Helper assist rules; upon reconnection, RSUs resynchronize and the controller resumes optimization. The specific control timing parameters used in our evaluation are detailed in Table 1.

First, a real-time RSU suitability estimator aggregates vehicular density, link quality, cache state, and resource availability to produce per-RSU scores that guide the PSO search. The cluster RSU Selection Module then employs Particle Swarm Optimization (PSO) to identify optimal cluster RSUs and assign roles (Leader, Helper, or Inactive) among candidate RSUs deployed across the network. Each particle encodes a role-assignment map and is evaluated by a fitness function that considers local traffic density, historical request patterns, RSU resource capacity, and network connectivity. The PSO search iteratively refines assignments to balance resource usage and reduce latency.

Once cluster RSUs are selected, the Replication Layer Management System establishes a content-replication structure across the selected RSUs and maintains it online. This component enables real-time distribution of requested content across sectors; replicas are adaptively pushed or pulled according to regional demand so that frequently accessed items remain locally available, thereby shortening paths and reducing transmission delay. Complementing this, the Adaptive Sectoring Mechanism allows each Leader RSU to adjust its communication radius and operational scope. Under high load, the Leader contracts its sector to prioritize nearby sessions; under light load, it may expand coverage to assist neighbors. This dynamic adjustment helps prevent void areas while preserving coverage continuity.

Finally, a load-adaptation routine monitors per-Leader load ratios against assist and fail thresholds, triggers association offload and selective Helper activation when limits are exceeded, and, if pressure persists, re-invokes PSO for role updates; during slack periods, underutilized nodes enter energy-saving modes while preserving coverage guarantees.

### 4.2. Real-Time RSU Suitability Estimation

To support adaptive optimization and real-time content distribution, each VANET node (vehicle or RSU) is modeled with a comprehensive state vector representing its operational parameters. This allows dynamic construction of a weighted graph, accurately reflecting network conditions at any given moment.

Let each node i∈V be represented by a multi-dimensional entity vector:

(3) $$E_{i}=\left[L_{i},C_{i},S_{i},B_{i},T_{i},E_{i},D_{i}\right]$$

Here, $L_{i}$ denotes the geographical location of node *i*, typically represented by GPS coordinates. The component $C_{i}$ reflects the available CPU resources at node *i*, while $S_{i}$ indicates the remaining storage capacity. $B_{i}$ represents the communication bandwidth currently available at node *i*, and $T_{i}$ captures the current traffic load experienced by the node. Additionally, $E_{i}$ signifies the residual energy level of node *i*, which is crucial for energy-aware optimization decisions. Lastly, $D_{i}$ specifies the content type or data being stored or served by the node, playing an essential role in content-aware routing and replication.

The network is thus modeled as a weighted undirected graph:

(4) G=(V,E,W)

where *V* is the set of active network nodes (vehicles and RSUs), *E* is the set of feasible bidirectional communication links, and *W* is the edge weight function reflecting link conditions.

An edge $e_{ij}\in E$ exists if the nodes *i* and *j* are within mutual communication range:

(5) $$e_{ij}\in E\Leftrightarrow \mathrm{dist}\left(L_{i},L_{j}\right)\leq \min\left(R_{i},R_{j}\right)$$

The edge weight $w_{ij}$ between nodes *i* and *j* is dynamically calculated using multiple parameters:

(6) $$w_{ij}=\alpha_{1}\;\cdot \;\frac{1}{B_{ij}}+\alpha_{2}\;\cdot \;T_{j}+\alpha_{3}\;\cdot \;\frac{1}{{\mathrm{LinkDuration}}_{ij}}+\alpha_{4}\;\cdot \;f\left(D_{i},D_{j}\right)$$

In this formulation, $B_{ij}$ refers to the effective bandwidth of the link connecting nodes *i* and *j*, and $T_{j}$ represents the traffic load at node *j*. The term ${\mathrm{LinkDuration}}_{ij}$ signifies the expected duration of the link between these two nodes, which affects connectivity stability. The function $f(D_{i},D_{j})$ quantifies the content relevance matching between nodes *i* and *j*, highlighting the suitability of content exchange. The parameters $\alpha_{k}$ are configurable weighting coefficients, enabling fine-tuning according to different network optimization goals. Real-time updates to $E_{i}$ allow the graph *G* to evolve, enabling topology-aware optimization, adaptive RSU coordination, dynamic sector partitioning, and intelligent replication strategies.

To dynamically evaluate each RSU’s suitability for selection as a Leader RSU or Helper RSU, we propose an enhanced scoring function that is sensitive to both resource availability and content-specific context. This function incorporates heterogeneous resources and the significance of the RSU’s cached content, addressing various demands arising from multimedia and sensor data services. Let the suitability score for RSU *i* be denoted by $f_{i}$, calculated as follows:

(7) $$f_{i}=\omega_{1}R_{i}^{\mathrm{cpu}}+\omega_{2}R_{i}^{\mathrm{storage}}+\omega_{3}R_{i}^{\mathrm{bw}}-\omega_{4}L_{i}^{\mathrm{traffic}}+\omega_{5}D_{i}^{\mathrm{degree}}$$

Here, $R_{i}^{\mathrm{cpu}}$ represents the normalized available CPU resources at RSU *i*, reflecting the current unused processing power. The term $R_{i}^{\mathrm{storage}}$ indicates the normalized remaining storage capacity, essential for data caching. The effective communication bandwidth between RSU *i* and its neighboring nodes is denoted by $R_{i}^{\mathrm{bw}}$. Conversely, $L_{i}^{\mathrm{traffic}}$ signifies the estimated traffic load at RSU *i*, where a higher value implies greater congestion. Finally, $D_{i}^{\mathrm{degree}}$ quantifies the uniqueness or distribution level of content held by RSU *i*, emphasizing the strategic importance of its stored data within the network.

The coefficients $\omega_{1}$ through $\omega_{5}$ are adaptive, content-aware weights adjusted according to the requested service type. For instance, video streaming services may prioritize $R_{i}^{\mathrm{bw}}$ and $R_{i}^{\mathrm{storage}}$ through increased weights, while real-time sensor data services may give precedence to $R_{i}^{\mathrm{cpu}}$ and $L_{i}^{\mathrm{traffic}}$. The weight vector $\vec{\omega }$ is defined as a function of the content category *c*:

(8) $$\vec{\omega }\left(c\right)=g\left(c\right)$$

The function g(c) dynamically adjusts resource weights based on the content type c∈{video,sensor,control,navigation}. Given two RSUs *i* and *j* with identical resource profiles but different content distributions ($D_{i}^{\mathrm{degree}}\neq D_{j}^{\mathrm{degree}}$), the resulting scores will differ if the provided coefficient $\omega_{5}$ is positive:

(9) $$f_{i}\neq f_{j}\;\mathrm{if}\;\omega_{5}>0$$

This scoring approach ensures content sensitivity and influences the Particle Swarm Optimization (PSO) fitness evaluation, directly impacting RSU role assignment decisions. The proposed mechanism ensures fairness, promoting the selection of RSUs with abundant and relevant resources while reducing load on units experiencing higher congestion.

### 4.3. PSO-Based RSU Role Assignment and Coverage Optimization

To ensure spatial balance in RSU role assignment and to prevent redundant coverage or service voids, the proposed framework derives a map-based sector partitioning mechanism as a foundational step. Before executing the PSO-based selection of Leader and Helper RSUs, the entire network topology is divided into logical geographic sectors, each of which functions as an independent optimization unit. In this approach, K-means clustering is applied to the geographical distribution of vehicles and RSUs to derive spatial sectors. Unlike uniform grid partitioning or density-based methods such as DBSCAN, K-means allows the system to define sector boundaries based on the actual node distribution while ensuring that a predetermined number of sectors is maintained. This not only simplifies coordination but also ensures consistent optimization scope for subsequent processing.

Each RSU is uniquely assigned to one of the resulting K-means clusters, forming the basis for sector-specific role allocation. Within each cluster, the PSO algorithm is independently executed to determine the most suitable Leader RSUs and their supporting Helper RSUs, considering localized traffic load, inter-RSU distance, and available resources. This localized optimization helps avoid excessive overlap between neighboring Leaders and enables balanced service distribution across the network.

To enhance the adaptability of the proposed cluster RSU framework in dynamic network environments, we design a Particle Swarm Optimization (PSO)-based mechanism to determine both the optimal number and spatial assignment of Leader and Helper RSUs. The key objective is to match the assignment of the infrastructure role with real-time vehicular traffic, network topology, and resource availability, while minimizing energy consumption and avoiding service bottlenecks. Let the candidate RSU set be denoted by $R=\{r_{1},r_{2},\ldots ,r_{N}\}$. For each RSU $r_{i}\in R$, its suitability score $f_{i}$ is computed using the multi-resource and content sensitive scoring function defined previously. The PSO algorithm seeks to find an optimal assignment of $K_{\mathrm{Leader}}$ Leader RSUs and $K_{\mathrm{Helper}}$ Helper RSUs that maximizes the collective utility of the network.

First, energy-deficient RSUs are excluded from the search space to preserve network longevity:

(10) $$\mathrm{If}\;E_{i}<E_{\min},\;\mathrm{then}\;r_{i}\notin R_{\mathrm{valid}}$$

Each particle in the PSO swarm encodes a solution vector:

$$\vec{x}=\left[K_{\mathrm{Leader}},K_{\mathrm{Helper}},\vec{l},\vec{h}\right]$$

where $K_{\mathrm{Leader}}$ and $K_{\mathrm{Helper}}$ represent the selected counts of Leader and Helper RSUs, and $\vec{l},\vec{h}$ are index vectors indicating their respective RSU assignments from $R_{\mathrm{valid}}$.

The fitness function is formulated as:

(11) $$F\left(\vec{x}\right)=\sum_{i\in \vec{l}}f_{i}+\lambda \sum_{j\in \vec{h}}f_{j}-\gamma \;\cdot \;\Psi \left(\vec{l},\vec{h}\right)$$

Here, λ controls the relative weight of Helper RSUs, while $\Psi (\vec{l},\vec{h})$ is a spatial penalty function defined as:

(12) $$\Psi \left(\vec{l},\vec{h}\right)=\sum_{\begin{array}{c} i,j\in \vec{l}\\ i\neq j \end{array}}\delta \left(d_{ij}<D_{\min}\right)+\sum_{i\in \vec{l},j\in \vec{h}}\delta \left(d_{ij}<R_{\min}\right)$$

where $d_{ij}$ is the Euclidean distance between RSUs *i* and *j*, and δ(·) is an indicator function returning 1 if the condition is satisfied, and 0 otherwise. $D_{\min}$ is the minimum separation distance enforced between Leader RSUs to avoid redundancy, and $R_{\min}$ restricts Helper RSUs from clustering too closely to their Leaders.

Upon convergence, the PSO yields an optimal configuration:

$$L^{*}=\vec{l},\;H^{*}=\vec{h}$$

satisfying:

$$\left|L^{*}\right|=K_{\mathrm{Leader}},\;\left|H^{*}\right|=K_{\mathrm{Helper}},\;\mathrm{and}\;F\left({\vec{x}}^{*}\right)=\max_{\vec{x}}F\left(\vec{x}\right)$$

This dynamic assignment procedure allows the cluster RSU system to autonomously adjust the number, role, and distribution of RSUs in response to spatiotemporal changes in network demand. By jointly considering fitness, spacing, and resource awareness, this approach ensures that infrastructure nodes are optimally placed and sufficiently distinct in role and location.

Following the selection of Leader RSUs, each Leader $r_{i}\in L$ serves as the center of a logical cluster RSU cell. The formation of each cell is based on the spatial proximity, link stability, and traffic load of neighboring RSUs. Formally, for each Leader RSU $r_{i}$, we define its cell $C_{i}$ as:

(13) $$C_{i}=r_{j}\in R|\mathrm{dist}\left(r_{i},r_{j}\right)\leq R_{\mathrm{init}}\wedge \rho_{ij}>\rho_{\min}\wedge T_{j}<T_{\mathrm{th}}$$

where $R_{\mathrm{init}}$ is the initial coverage radius, $\rho_{ij}$ is the normalized link reliability between $r_{i}$ and $r_{j}$, and $T_{j}$ is the current traffic load at RSU $r_{j}$. This formulation ensures that each cell initially includes RSUs that are close, stably connected, and lightly loaded, thereby promoting balanced and efficient content dissemination.

To optimize the coverage areas of each cluster RSU cell in response to dynamic traffic distribution and vehicular density, we employ a secondary PSO routine. This mechanism adjusts the radius $R_{i}$ of each cell $C_{i}$ to minimize overlap between neighboring cells and balance the served load.

The objective function to be minimized is:

(14) $$F=\lambda_{1}\;\cdot \;O_{\mathrm{total}}+\lambda_{2}\;\cdot \;\sigma_{T}+\lambda_{3}\;\cdot \;\sigma_{N}$$

where $O_{\mathrm{total}}$ denotes the total overlapping coverage area among all cluster RSU cells, which reflects redundancy and resource waste due to spatial overlap. The term $\sigma_{T}$ represents the standard deviation of traffic load among all cluster RSU cells, serving as a metric for load imbalance. Likewise, $\sigma_{N}$ denotes the standard deviation in the number of vehicles served per cell, quantifying disparities in service distribution. The coefficients $\lambda_{1}$, $\lambda_{2},\lambda_{3}$ are tunable weighting parameters

Each particle in this PSO search represents a vector $\left[R_{1},R_{2},\ldots ,R_{K_{\mathrm{Leader}}}\right]$ of candidate coverage radii. The update rule follows standard PSO mechanics, with fitness evaluated using the objective function *F*. By minimizing *F*, the PSO algorithm finds a balanced partitioning that avoids both congestion and underutilization, thus achieving scalable and adaptive coverage management across the VANET environment.

### 4.4. Replication Layer Coordination and Request Routing

As described in Section 4.1, the Replication Layer is orchestrated by the MEC-based controller using periodic control-plane updates. Once the cluster RSU infrastructure is established through the selection and spatial organization of Leader RSUs, the proposed system proceeds to form a global overlay structure called the Replication Layer. This virtual layer is collaboratively constructed by all active Leader RSUs. Each cluster RSU contributes its available computational, storage, and bandwidth resources to the shared layer, allowing for centralized yet dynamically adaptive management of distributed content delivery operations.

**Figure 2:**
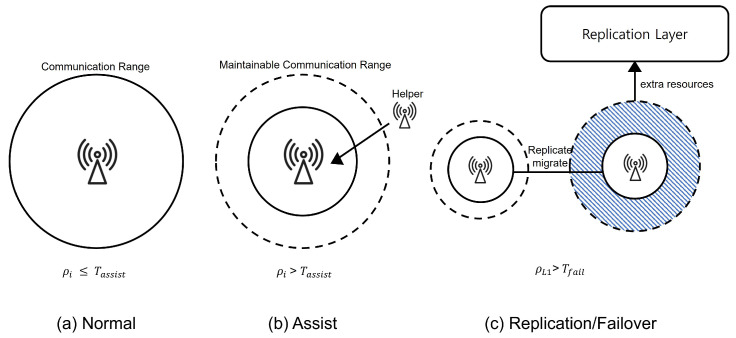
Replication Layer coordination under load.

Figure 2 illustrates the staged adaptation of PDRA under increasing load. In Figure 2a, when the per-RSU utilization $\rho_{i}\leq T_{\mathrm{assist}}$, the Leader operates at its nominal communication range and serves all associated vehicles. In Figure 2b, once $\rho_{i}>T_{\mathrm{assist}}$, the Leader contracts its sector toward a maintainable communication range (outer dashed circle) to protect latency for nearby users, while a neighboring node designated as a Helper is activated to assist with overflow traffic at the boundary. In Figure 2c, if the Leader’s load continues to escalate and exceeds the failover threshold (e.g., $\rho_{L1}>T_{\mathrm{fail}}$), the Replication Layer allocates extra resources and replicates or migrates popular content to a neighboring cluster. Subsequent requests are routed to that replica until pressure subsides. These transitions realize the “assist → replication/failover” policy while preserving coverage continuity and preventing void areas.

Formally, let $G=r_{1},r_{2},\ldots ,r_{K_{\mathrm{Leader}}}$ represent the set of all active cluster RSUs. The Replication Layer is modeled as an abstract overlay graph $G\mathrm{rep}=(G,E_{\mathrm{rep}})$ where edges $E_{\mathrm{rep}}$ represent control-plane communication links and coordination agreements. Each node $r_{i}\in G$ maintains a registry of its current resource state:

(15) $$R_{i}=\left[R_{i}^{\mathrm{cpu}},R_{i}^{\mathrm{storage}},R_{i}^{\mathrm{bw}},T_{i},D_{i}\right]$$

This allows the Replication Layer to maintain a global view of the distributed resource availability and content distribution, which is essential for coordinating dynamic content replication and cluster RSU task delegation.

When a vehicle node generates a content request, the system leverages the Replication Layer to dynamically assign the optimal cluster RSU for request handling. This decision is based on a joint consideration of the requester’s location, the type of content requested, and the real-time status of all cluster RSUs in terms of load and available resources. Let a vehicle $v_{k}$ submit a request $q_{k}=\left(L_{k},c_{k}\right)$, where $L_{k}$ denotes the vehicle’s location and $c_{k}$ represents the content type. The optimal cluster RSU $r^{*}$ is selected by solving:

(16) $$r=\arg\min_{r_{i}\in G}\left[\delta \left(L_{k},L_{i}\right)+\beta_{1}\;\cdot \;\phi_{i}\left(c_{k}\right)+\beta_{2}\;\cdot \;T_{i}\right]$$

where $\delta (L_{k},L_{i})$ is the physical or communication distance between the vehicle and cluster RSU $r_{i}$, $\phi_{i}\left(c_{k}\right)$ is a content cost function representing the effort required for RSU $r_{i}$ to deliver content type $c_{k}$, and $T_{i}$ is the current traffic load at $r_{i}$. Coefficients $\beta_{1}$ and $\beta_{2}$ balance the relative importance of content locality and load balancing.

This optimization ensures that the selected cluster RSU can serve the request efficiently while minimizing latency and preventing overload. By maintaining global resource visibility, the Replication Layer plays a key role in enabling intelligent, real-time coordination across the distributed RSU infrastructure.

### 4.5. Load Adaptation and Energy-Aware RSU Reconfiguration

To ensure robust system performance under dynamic traffic surges and resource constraints, we propose a three-stage load adaptation mechanism integrated into the Replication Layer. This mechanism reacts to changes in RSU conditions based on pre-defined threshold parameters.

**Stage 1: Load Assist Activation.** When the load ratio $\rho_{i}$ of a cluster RSU $r_{i}$ exceeds a predefined assist threshold $T_{\mathrm{assist}}$, i.e.,

(17) $$\rho_{i}>T_{\mathrm{assist}},$$

the RSU initiates a local recovery protocol by reducing its coverage radius and offloading excess load to nearby cluster RSUs. This offloading is coordinated through the Replication Layer, enabling cooperative support from less-utilized neighboring RSUs.

**Stage 2: Helper RSU Activation.** This second stage is escalated by the proposed framework, if the load $\rho_{i}$ continues to increase and surpasses a failure threshold $T_{\mathrm{fail}}$:

(18) $$\rho_{i}>T_{\mathrm{fail}}.$$

In this case, pre-designated Helper RSUs in the vicinity are activated to absorb and distribute the excess processing and transmission demands. This step is proactive and aims to avoid congestion or packet loss in high-density vehicular scenarios.

**Stage 3: Leader Reassignment and cluster RSU Reorganization.** In scenarios where the high load persists and RSU $r_{i}$ shows signs of long-term degradation (e.g., declining energy or resource availability), the PSO algorithm is re-invoked. A new Leader RSU is selected using the same scoring and optimization criteria as before, and the cell previously served by $r_{i}$ is reassigned:

(19) $$\begin{array}{c} \left(\rho_{i}>T_{\mathrm{fail}}\right)\;\wedge \;\left(\Delta_{\tau }R_{i}^{\mathrm{cpu}}<0\;\wedge \;\Delta_{\tau }R_{i}^{\mathrm{storage}}<0\right)\;\Rightarrow \\ \mathrm{PSO}\;\mathrm{re}\text{-}\mathrm{execution} \end{array}$$

where $\Delta_{\tau }X$ denotes the change in *X* over the past window τ (current value minus the value τ time units ago), i.e., the current value of *X* minus its value *W* time units ago. This final stage enables long-term network stability and maintains service continuity by adaptively refreshing the cluster RSU structure. Together, this multi-stage mechanism provides resilience and flexibility in handling traffic fluctuations and RSU degradation, ensuring that the VANET remains robust and responsive under various dynamic changes and stresses.

To improve overall network sustainability and reduce unnecessary power consumption, the proposed framework implements an energy-aware RSU role optimization policy. This mechanism is triggered during periods of low demand or when RSUs are underutilized. The system continuously monitors the operational status of all active Leader and Helper RSUs. When an RSU’s utilization—defined by its CPU, bandwidth, and traffic load—falls below a minimal activity threshold $U_{\min}$ for a predefined time window τ, it becomes a candidate for deactivation. Formally, for RSU $r_{i}$:

(20) $$\mathrm{If}\;U_{i}\left(t\right)<U_{\min}\;\forall t\in \left[t_{0},t_{0}+\tau \right],\;\mathrm{then}\;r_{i}\to \mathrm{Sleep}\;\mathrm{Mode}$$

where $U_{i}\left(t\right)$ is the composite utilization index of RSU $r_{i}$ at time *t*. Inactive or low-priority Helper RSUs are first targeted for sleep transition. If energy-saving thresholds remain unmet, redundant Leader RSUs—determined based on overlap in coverage and low request frequency—are also evaluated for deactivation. The selection preserves service continuity by ensuring that at least one cluster RSU remains active per logical region. The Replication Layer enforces a global policy of minimal necessary RSU activation, maintaining just enough coverage and resource availability to satisfy current demand. It dynamically reawakens sleeping RSUs when usage spikes are detected or requests exceed the current infrastructure’s capability. This dynamic sleep scheduling mechanism contributes to long-term network longevity, especially in scenarios involving RSUs with limited power sources (e.g., solar-powered units), and enhances energy efficiency without compromising content delivery performance.

## 5. Performance Evaluation

In this section, we evaluate the performance of the proposed framework through simulation results. We first provide environments and parameters for conducting our simulations and next explain simulation results for evaluating the performance of the proposed framework compared with the existing protocols.

### 5.1. Simulation Environment and Parameter Settings

We evaluate the proposed framework on ns-3.43 using a 5 km × 5 km urban map of Seattle downtown imported from OpenStreetMap (OSM). The road graph and intersections are taken directly from OSM so that vehicle mobility is constrained to real road segments. We deploy 1000 vehicles whose speeds are uniformly distributed in [30 km/h,60 km/h] and simulate 600 s per run. Wireless access uses IEEE 802.11p [38] operating at 5.9 GHz with a link bandwidth of 10 Mbps.

For infrastructure, 200 RSUs are provisioned on the road network. The control plane operates in a periodic loop with a state reporting interval of $\Delta T_{\mathrm{state}}=1\;s$ and a PSO re-optimization interval of $\Delta T_{\mathrm{control}}=5\;s$. To ensure infrastructure stability under mobility, a minimum holding time $T_{\mathrm{hold}}=10\;s$ is applied to RSU role assignments. At runtime, the PSO algorithm (configured with 30 particles and 100 iterations) dynamically selects Leader and Helper RSUs based on load thresholds ($T_{\mathrm{assist}}=0.8$, $T_{\mathrm{fail}}=0.95$). The RSU communication radius is adaptive around a nominal 400 m to balance coverage and interference.

Application traffic consists of mixed content requests with a fixed composition of 30% video and 70% sensor data. The application data unit for sensor updates is 1 KB, while each video request uses a 5 MB data unit. Content utility scores are calculated using distinct weight vectors ($\omega_{1}\ldots \omega_{5}$) as detailed in Table 1, prioritizing latency for sensor data and bandwidth/storage for video services. All detailed simulation parameters, including PSO constants and utility weights, are summarized in Table 1. Each experiment is repeated 30 times with independent random seeds, and we report the mean values with confidence intervals.

### 5.2. Computational Complexity and Runtime Analysis

To validate the real-time feasibility of the proposed PDRA framework, we analyzed the computational complexity and measured the actual execution time of the optimization module.

#### 5.2.1. Complexity Analysis

The core of the PDRA framework relies on Particle Swarm Optimization (PSO). The computational complexity of PSO is generally given by O(I·P·D), where *I* is the maximum number of iterations, *P* is the population size (number of particles), and *D* is the dimensionality of the search space. However, PDRA employs K-means clustering to partition the global network into *K* independent sectors. Consequently, the optimization is executed locally within each sector, reducing the effective dimensionality to $D\approx N_{total}/K$, where $N_{total}$ is the total number of RSUs. This sector-based decomposition ensures that the algorithm scales efficiently even in large-scale dense networks.

#### 5.2.2. Actual Runtime and Sensitivity

We measured the average execution time of the PSO algorithm on a standard computing node equipped with an Intel Core i7-13700K CPU and 32 GB RAM. Table 2 summarizes the impact of PSO hyperparameters (*P* and *I*) on both solution quality (normalized fitness gain) and execution time.

As shown in Table 2, increasing the search space from (P=20,I=50) to (P=50,I=200) improves the fitness value but significantly increases computational cost. We observed that the configuration of P=30 and I=100 offers the best trade-off, achieving a 12% performance gain over the baseline parameters with a modest runtime of approximately 42.1 ms per sector. Given that the control plane update interval is $\Delta T_{\mathrm{control}}=5$ s (5000 ms), the computation time accounts for approximately 0.84% of the total control epoch. This confirms that the PDRA algorithm is lightweight enough to operate in real-time on edge servers without introducing significant processing latency.

### 5.3. Simulation Results

We evaluated the proposed framework (PDRA) against representative baselines in the environment summarized in Table 1 and the accompanying setup paragraph. The baselines capture complementary design choices: Static-RSU (no role selection or replication), AALB (application-aware RSU load balancing), HRL-PC (proactive caching in a vehicle–edge–cloud hierarchy), PSUV (PSO-assisted delivery). All schemes adhere to the same wireless stack, workload composition, cache/replication budgets, and control-plane update period; method-specific hyperparameters were tuned by grid search within equal budgets and disclosed with the results.

To ensure the reproducibility of our comparative evaluation, we explicitly summarize the observability and control scope of each baseline method.

#### 5.3.1. Real-Time Feasibility Analysis

To validate the feasibility of PSO-based reconfiguration in dynamic VANETs, we compare the computational latency with the network coherence time. As shown in Table 2, the average execution time of the PSO algorithm is 42.1 ms on a standard edge server. In contrast, given a vehicle speed of 60 km/h and an RSU coverage radius of 400 m, the average residence time (coherence time) of a vehicle within an RSU’s range is approximately 24 s. Since the computation time ( 42.1 ms) is significantly shorter than the topology change interval (≈24 s) and the control period ($\Delta T_{control}=5$ s), the optimized roles and radii are applied well before the network topology becomes obsolete, ensuring real-time stability.

#### 5.3.2. Control Plane Overhead

We also evaluated the signaling overhead generated by global synchronization. The control messages, which include RSU load states and Bloom-filter-based cache digests, are approximately 2 KB in size. With a reporting interval of $\Delta T_{state}=1$ s, the aggregate control traffic consumes less than 1.5% of the total backhaul bandwidth, even under peak loads where data traffic (e.g., 5 MB video chunks) dominates. This confirms that the proposed centralized coordination does not induce signaling storms.

- **Static-RSU:** Represents a fixed-infrastructure baseline without role reassignment, RSU radius adaptation, or inter-RSU replication.
- **AALB [26]:** Represents an application-aware load balancing approach that reallocates workloads among RSUs based on monitored states (e.g., response time, utilization, and application deadlines). However, it does not perform physical radius control nor content replication.
- **HRL-PC [19]:** Represents a proactive caching scheme in a vehicle–edge–cloud hierarchy. Vehicles request data from the nearest RSU, and cache misses are fetched from the cloud. An HRL-based policy proactively updates cached items at RSUs, but it operates without radius control and without explicit RSU-to-RSU cooperative replication.
- **PSUV [25]:** Represents PSO-assisted delivery decisions at the routing level considering mobility, but it does not include infrastructure-level role or radius control.

Table 3 summarizes the detailed observation scope and control knobs of all compared methods.

Performance is reported using standard QoS and network efficiency indicators. We measure packet delivery ratio, end-to-end delay (mean and 95th percentile), jitter, throughput, control-plane overhead, Jain’s fairness of RSU utilization, coverage ratio, and core backhaul traffic. Additionally, we evaluate the total energy consumption to validate the sustainability of the proposed infrastructure sleep mode. Curves present means with 95% confidence intervals over independent repetitions; significance is tested with Welch’s *t*-test (or Mann–Whitney *U* upon normality rejection) with Holm correction.

To exercise distinct stressors without altering the environment, we vary the concurrent session count (load sensitivity of delivery and latency), sweep packet error rate or SINR (channel robustness), change vehicle speed and density (coverage, voids, and handover stability), inject popularity shocks by replacing a large fraction of the top-*K* video items and relocating hotspots at predefined times (cache-hit recovery time and backhaul spikes), and align traces around handover events (±20 s) to quantify session continuity. This design isolates the specific contributions of PDRA—PSO-based Leader/Helper selection, adaptive RSU range control, and a Replication Layer that localizes demand—against principled alternatives in load balancing, proactive caching, generic PSO-assisted delivery, and mobility-aware edge formation, while accounting for the operational overhead associated with each gain.

**Figure 3:**
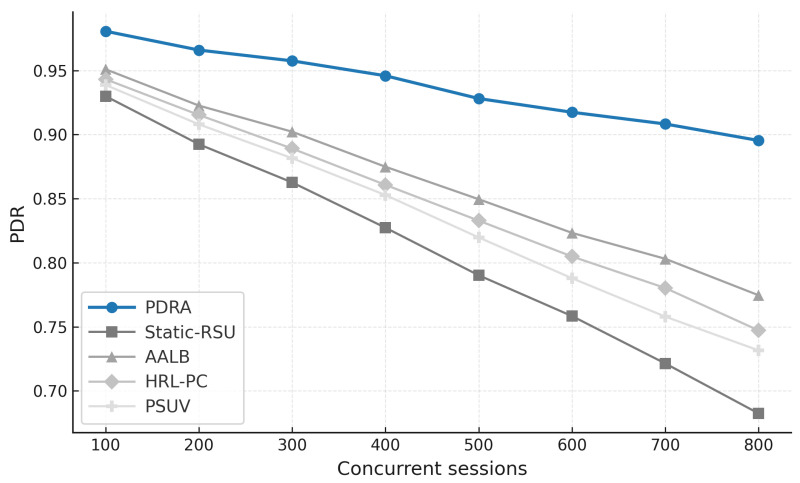
PDR vs. Sessions.

Figure 3 shows the packet delivery ratio (PDR) as a function of the number of concurrent sessions. Across all schemes the PDR declines as load increases because contention grows and RSU queues fill, which leads to collisions and buffer drops. PDRA shows the slowest decay. It distributes associations through PSO-based Leader-Helper selection, and it adapts the RSU radius to tighten coverage in dense blocks and to avoid voids in sparse areas. The Replication Layer places popular content at Leaders and serves nearby requests via Helpers, which shortens paths and reduces retransmissions in congested cells. These choices limit loss propagation over multi-hop routes, so PDRA maintains a clear margin in the high-load regime.

The baselines fall for different reasons. Static-RSU lacks role selection and range control, so traffic concentrates on a few hotspots and both backoff and queue drops increase rapidly. PSUV uses PSO for delivery decisions but does not partition load at the infrastructure level, which leaves paths longer on average and forces flows through busy cells where losses accumulate. AALB redistributes traffic using application awareness, yet without replication and range adaptation it cannot prevent local saturation when concurrency is high. HRL-PC benefits from proactive caching and therefore performs better than Static-RSU, PSUV, and AALB under moderate load, but the absence of per-epoch role reconfiguration means cache placement does not always coincide with balanced channel access, so reliability still degrades as sessions scale up. Overall, reliability is determined less by the forwarding rule itself and more by how effectively a scheme shapes contention and balances RSU queues, and PDRA is designed to do exactly that.

**Figure 4:**
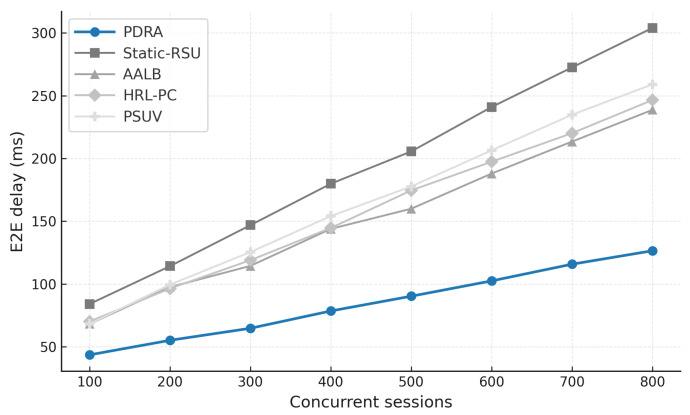
Delay vs. Sessions.

Figure 4 shows the average end-to-end delay as the number of concurrent sessions increases. All schemes exhibit rising delay with load because contention grows and RSU queues lengthen, increasing backoff and service time. However, PDRA scales more gracefully due to three key mechanisms. First, the PSO-based Leader–Helper selection spreads associations away from hotspots. Second, the adaptive RSU radius keeps collision domains small in dense areas while avoiding coverage holes in sparse areas. Finally, near-demand replication shortens paths so packets bypass congested relays.

Quantitatively, PDRA achieves substantially lower delay than the baselines over the entire range. Averaged across all loads, PDRA reduces the mean delay by approximately 45% compared with the best baseline (AALB) and over 50% compared with the baseline average. The advantage is most visible at high load. For instance, at 800 sessions, the delay of PDRA is approximately 126 ms, whereas the best competitor records approximately 239 ms. Similarly, at 700 sessions, PDRA achieves 116 ms compared to 213 ms for the baselines. Static-RSU grows fastest because it lacks role selection and range control. PSUV retains longer routes since infrastructure-level load partitioning is absent. Although AALB and HRL-PC improve over Static-RSU, they still incur larger queueing delays when many sessions concentrate on the same access domain.

**Figure 5:**
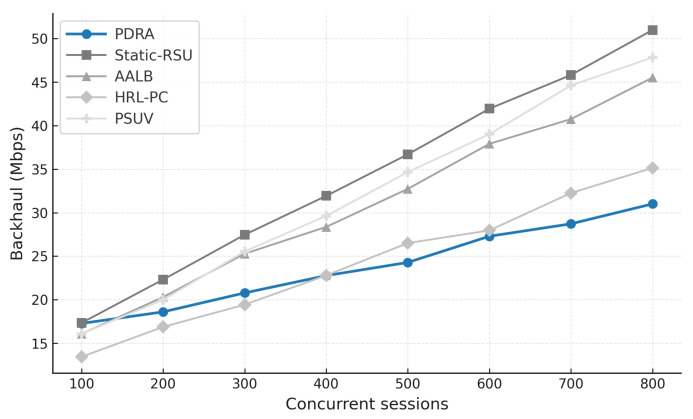
Backhaul vs. Sessions.

Figure 5 shows how core backhaul traffic grows with the number of concurrent sessions. All schemes increase core-bound traffic as load rises because cache misses become more frequent and more packets traverse Cluster RSUs. PDRA maintains the lowest backhaul traffic in the high-load region by combining PSO-based Leader–Helper selection with an adaptive RSU radius and near-demand replication. Since popular content is placed at Leaders and served by nearby Helpers, a significant portion of requests is handled locally, avoiding multi-hop forwarding to the core.

At low load, HRL-PC can be slightly lower owing to aggressive proactive caching; however, beyond 500 sessions, PDRA dominates the performance. Averaged over the high-load points (≥500 sessions), PDRA reduces backhaul traffic by 8–9% relative to the best baseline (HRL-PC) and by 28% relative to the baseline average. Static-RSU increases fastest due to the lack of role selection and range control, which concentrates flows on a few gateways. PSUV does not partition load at the infrastructure level, so many requests still take long routes to a small set of egress RSUs. While AALB redistributes sessions per application, it cannot prevent local saturation and the resulting core-bound traffic because it lacks replication and radius adaptation capabilities.

**Figure 6:**
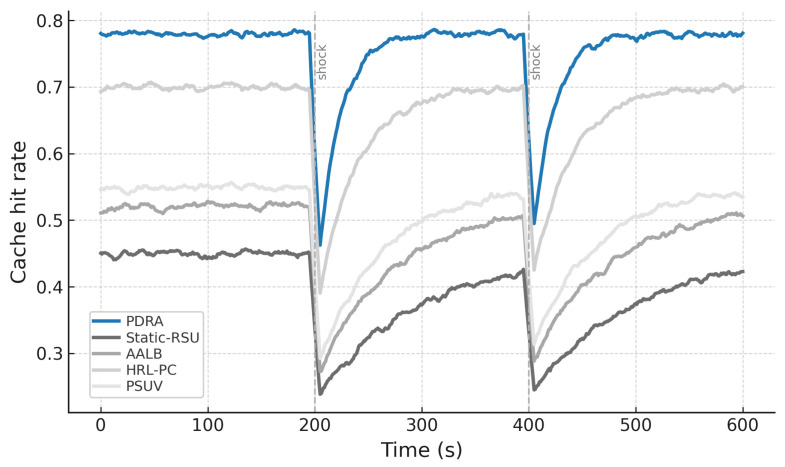
Hit rate vs. Time multi.

**Figure 7:**
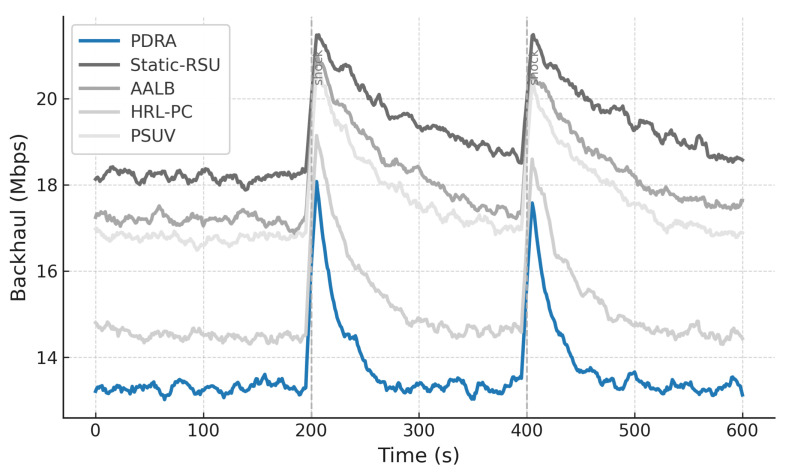
Backhaul vs. Time multi.

Figure 6 and Figure 7 show system behavior under two popularity shocks. At 200 s and 400 s, we replace a large share of the top-*K* items and relocate the hotspot so that both content and traffic locality change abruptly. Two shocks are used to assess not only how quickly each method adapts but also whether it adapts consistently across repeated disturbances. For the cache, we report the recovery time, defined as the time it takes for the hit rate to return to 95% of its pre-shock level. For the backhaul, we report the peak immediately after each shock and the spike duration until the curve falls back to within 10% of the pre-shock baseline.

PDRA adapts faster and yields smaller transients than all baselines. Its cache-hit recovery times are 46 s at 200 s and 43 s at 400 s, whereas the best baseline (HRL-PC) requires 78 s and 74 s. Averaged over both shocks, PDRA shortens recovery by 42%. The steady-state hit rate before each shock is also higher for PDRA (0.776) than for HRL-PC (0.696), an eight percentage-point gap. Backhaul peaks after the shocks are 17.96 Mbps and 17.48 Mbps with PDRA, compared with 19.07 Mbps and 18.61 Mbps for HRL-PC, a 6% reduction. The spike durations are 32 s and 27 s with PDRA versus 43 s and 41 s with HRL-PC, an average reduction of 30%. Even in steady state, the backhaul remains lower for PDRA ( 13.36 Mbps versus 14.56 Mbps, 8% lower). These effects follow from PSO-based Leader–Helper reassignment, RSU radius adaptation that aligns coverage with demand, and near-demand replication that places popular items where sessions originate, allowing cache locality and channel contention to realign promptly after each shock.

**Figure 8:**
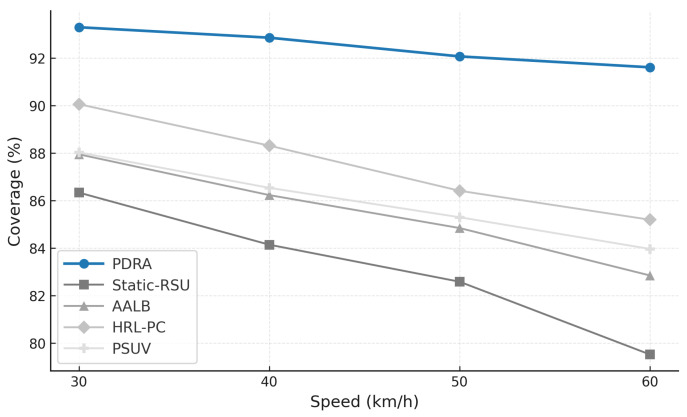
Coverage vs. Vehicle speed.

**Figure 9:**
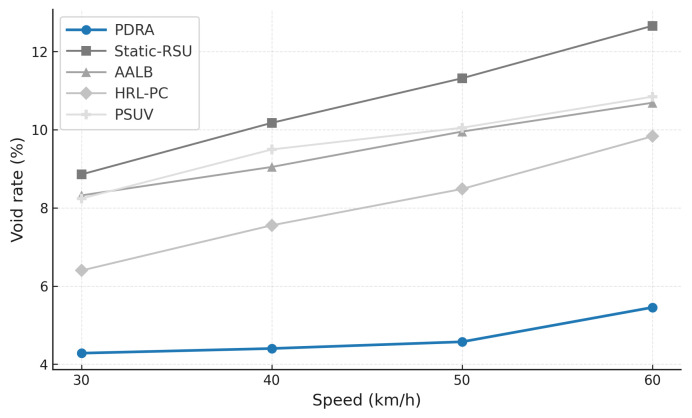
Void rate vs. Vehicle speed.

**Figure 10:**
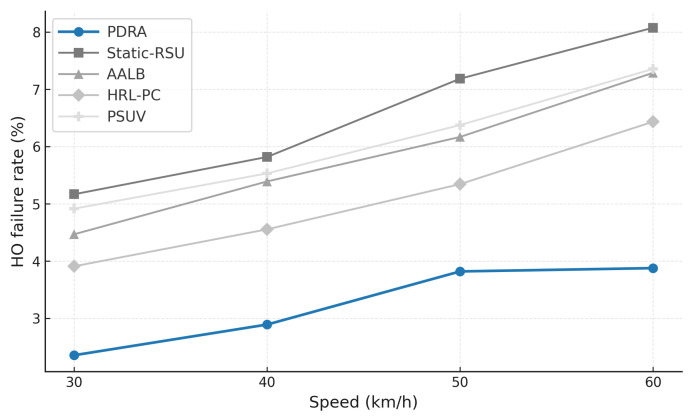
Handover (HO) failure rate vs. Vehicle speed.

Figure 8, Figure 9 and Figure 10 show how increasing vehicle speed from 30 km/h to 60 km/h affects coverage, voids, and handover robustness. All methods lose coverage and experience more voids and handover failures at higher speeds because vehicles dwell for a shorter time within each RSU footprint and the overlap window between adjacent RSUs narrows. This reduces the time available for association and handover preparation and amplifies contention near cell edges.

PDRA remains the most stable across all three metrics. In Figure 8, its coverage declines gently and stays above all baselines at every speed. The gap to the best competitor (HRL-PC) is 3–6 percentage points (averaging four percentage points), and the gap to the baseline average is 5–8 percentage points. In Figure 9, PDRA yields the lowest void rate throughout. Averaged over the four speeds, the void rate is lower by 40–45% relative to HRL-PC and by 50% relative to the baseline average, with the advantage widening at 60 km/h. In Figure 10, PDRA attains the lowest handover failure rate, reducing failures by 35% versus the strongest baseline and by 40–45% versus the baseline average.

These trends follow from the same design principles used in the load experiments. PSO-based Leader–Helper selection balances associations so edge cells do not saturate. The adaptive RSU radius increases controlled overlap in fast segments while avoiding overcoverage in slow segments, thereby lengthening the effective handover window without inflating contention domains. Near-demand replication and helper relaying keep most sessions local and shorten routes away from unstable edges. Together, these mechanisms sustain coverage, suppress voids, and improve handover reliability as speed rises.

Alongside PDR and delay, we evaluate Jain’s fairness because balanced RSU utilization is a first-order determinant of reliability and latency. When a few gateways become hotspots, collision probability and queueing delay rise; packet drops and tail latency follow, degrading overall QoS even when average throughput appears acceptable. A fairness metric shows whether an algorithm prevents such hotspots and benefits all sessions rather than shifting load to a small subset of RSUs. We adopt Jain’s index because it is bounded in [0,1], scale-invariant with respect to total demand, and comparable across loads and topologies, which makes it suitable for our setting.

**Figure 11:**
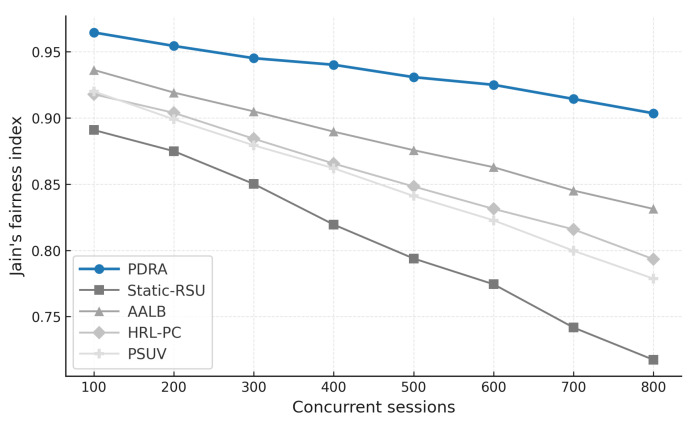
Fairness vs. Sessions.

Figure 11 plots Jain’s fairness as the number of concurrent sessions increases. All schemes become less fair at higher load as contention concentrates on a few Cluster RSUs and queues lengthen. PDRA degrades slowest and maintains the highest fairness across the entire range. Averaged over all loads, PDRA improves fairness by 6% relative to the best baseline (AALB) and by 11% relative to the baseline average. At 800 sessions, PDRA reaches 0.91 versus 0.83 for the best competitor. The total drop from 100 to 800 sessions is 0.055 for PDRA, whereas the best baseline drops by 0.10. These gains stem from PSO-based Leader–Helper selection that balances associations, adaptive RSU radius control that limits collision domains in dense areas, and near-demand replication that keeps flows local and prevents hotspot amplification.

**Figure 12:**
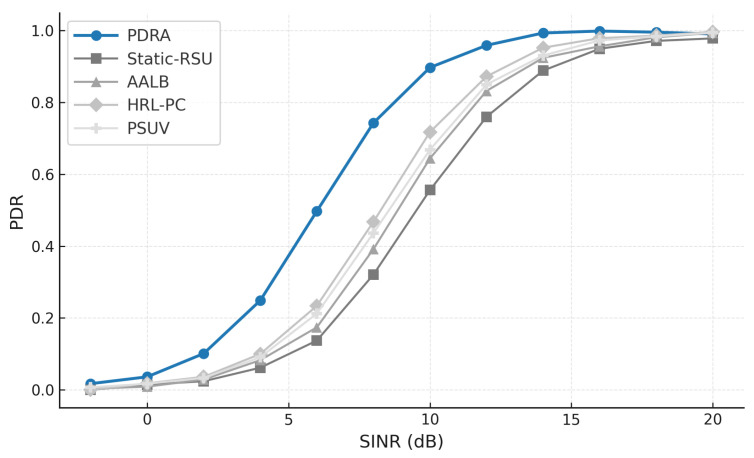
PDR vs. SINR.

Figure 12 shows the packet delivery ratio as a function of SINR with the MCS and frame length held fixed. The curves exhibit the expected S-shaped profile, and higher SINR yields higher reliability. PDRA is consistently left-shifted relative to all baselines. It reaches a PDR of 0.90 at 10 dB, whereas the best baseline (HRL-PC) requires 12 dB and Static-RSU needs 15 dB. Thus, PDRA lowers the SINR threshold for 0.90 delivery by about 2 dB versus the best competitor and by 5 dB versus Static-RSU. Averaged over the transition region 6 dB to 14 dB, PDRA delivers 10 percentage points higher PDR than the best baseline and 13 percentage points higher than the baseline average. Representative points follow the same trend: at 8 dB, PDRA attains 0.75 while the best baseline yields 0.46; at 12 dB, PDRA is 0.96 versus 0.93. These gains arise from PSO-based Leader–Helper selection that balances associations, adaptive RSU radius control that lowers contention in dense cells, and near-demand replication that shortens routes so packets traverse fewer error-prone hops, which together improve effective link robustness at a given SINR.

**Figure 13:**
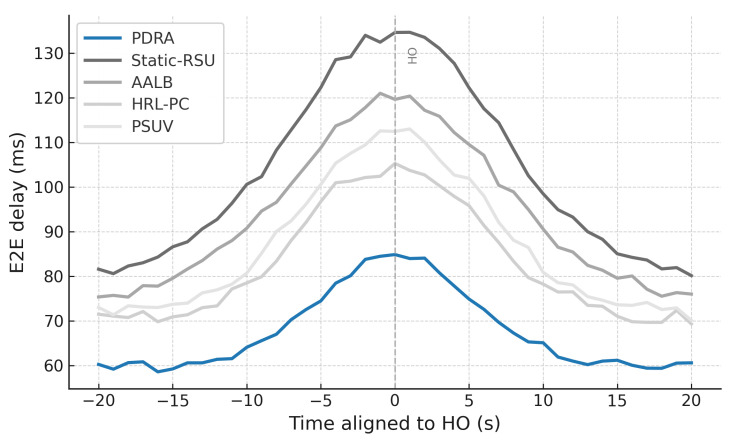
HO delay profile.

Figure 13 shows the end-to-end delay profile aligned to handover events, where τ=0 marks the association switch and the window spans ±20 s. All schemes exhibit a delay pulse around τ=0 because probing, re-association, and route updates increase service time and contention near cell edges. PDRA produces the smallest pulse and returns to its baseline fastest. The pre-HO baseline delay (e.g., τ∈[−20,−10]s) is 60 ms for PDRA versus 74 ms for the best baseline (HRL-PC), a 19% reduction. The peak at the handover instant is 86 ms for PDRA compared with 106 ms for HRL-PC and 135 ms for Static-RSU, i.e., reductions of 19% and 36%, respectively. The time to fall back to within 10% above the pre-HO baseline is 9 s for PDRA and 12 s for HRL-PC, a 25% shorter transient.

These improvements follow from three elements that act during handover. PSO-based Leader–Helper selection prevents edge saturation so queue growth around the boundary is limited. The adaptive RSU radius maintains controlled overlap, which enlarges the effective handover window without inflating collision domains. Helper relaying and near-demand replication keep sessions local during the switch, which shortens paths and masks short routing gaps. Together, these mechanisms lower the baseline delay, reduce the HO peak, and shorten the recovery interval.

**Figure 14:**
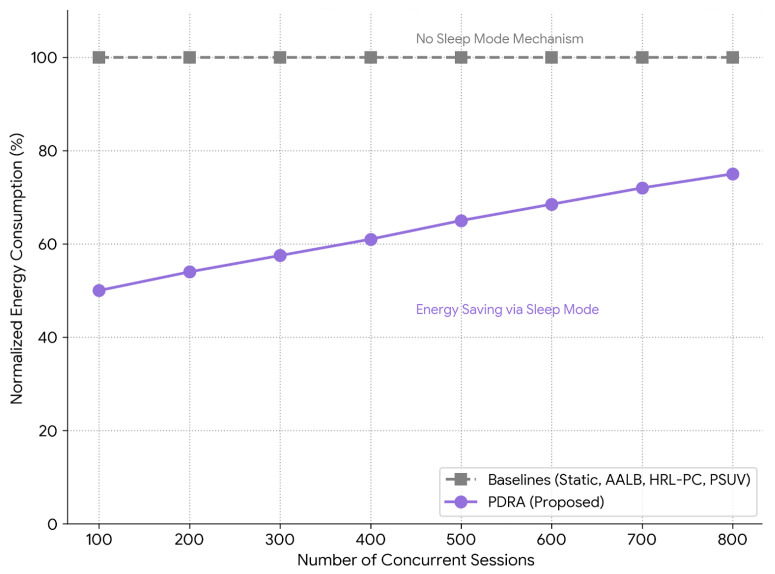
Normalized total energy consumption vs. traffic load. PDRA reduces energy usage by activating RSUs adaptively via Sleep Mode, whereas baselines maintain 100% activity.

Figure 14 shows the normalized total energy consumption of the network as the number of concurrent sessions increases. Since the baseline schemes (Static-RSU, AALB, HRL-PC, and PSUV) do not support dynamic infrastructure sleeping, they maintain all 200 RSUs in the active state, resulting in constant maximum energy consumption (100%). In contrast, PDRA utilizes the Sleep Mode mechanism to dynamically activate only the necessary Leader and Helper RSUs. As shown in the figure, PDRA achieves significant energy savings, reducing consumption by approximately 50% at low loads (100 sessions) and maintaining 25–30% savings even at high loads, thereby validating its energy-aware design.

## 6. Conclusions

This paper presented PDRA, a PSO-driven dynamic RSU role assignment framework for urban VANETs. The design fuses three mechanisms that act in concert on reliability, latency, and core load: PSO-based Leader–Helper selection that redistributes associations, adaptive RSU radius control that shapes collision domains while preserving coverage, and near-demand replication that aligns content locality with session locality. The key originality is the joint optimization of *roles, coverage, and replication* within one control loop, rather than optimizing only routing, only caching, or only static provisioning. We also adopt evaluation scenarios that reflect operational stressors—load sweeps, popularity shocks, mobility sweeps, SINR sweeps with fixed MCS and frame length, and handover-aligned delay profiles—so both steady-state and transient behavior are exposed.

Across all settings, PDRA delivers consistent gains. Under increasing concurrency, it lowers average end-to-end delay by about 45% relative to the best baseline and by 50% relative to the baseline average, while sustaining higher PDR in the high-load region. In the same regime, it cuts core backhaul by 8–9% versus the best baseline and by 28% versus the baseline average. Under popularity shocks, it restores the cache hit rate 42% faster, limits backhaul peaks by 6%, and shortens spike duration by 30%. As vehicle speed rises from 30 km/h to 60 km/h, PDRA maintains higher coverage, reduces the void rate by 40–45% with respect to the best baseline, and lowers the handover failure rate by 35%. Jain fairness also remains higher, improving by 6% over the best baseline and by 11% over the baseline average. In SINR sweeps, PDRA reaches approximately 0.90 PDR at roughly 2 dB lower SINR than the best competitor. Handover-aligned delay profiles show a lower baseline, a smaller peak at the switch, and a faster return to steady state.

In summary, PDRA upgrades urban vehicular networking by coordinating where vehicles attach, how coverage is shaped, and where content is placed. This unified control translates into higher reliability, lower delay, better fairness, and reduced core load under mixed video and sensor traffic, demonstrating that joint role, coverage, and replication optimization is a powerful lever for city-scale VANETs.

## Figures and Tables

**Table 1:** Simulation parameters and algorithm constants.

Parameter	Value
**Network and Traffic Scenario**	
Simulated network field	5000 m × 5000 m (Seattle downtown map)
Number of intersections	Based on real OpenStreetMap data
Number of roads	Realistic urban topology (OSM-derived)
Vehicle numbers	1000
Mobility model	Random road-constrained mobility
Speed of vehicles	30 km/h to 60 km/h
RSU numbers	200 (PSO dynamically selects Leaders/Helpers)
Communication range of RSUs	400 m (Adaptive via PSO)
MAC protocol	IEEE 802.11p
Frequency band	5.9 GHz
Link bandwidth	10 Mbps
Packet size	1 KB (Sensor data), 5 MB (Video)
Content request types	30% Video/70% Sensor data
**Control Plane Timing**	
State reporting period ($\Delta T_{\mathrm{state}}$)	1 s
Optimization/Commit period ($\Delta T_{\mathrm{control}}$)	5 s
Minimum holding time ($T_{\mathrm{hold}}$)	10 s
Simulation time	600 s
**PSO Hyperparameters**	
Inertia weight (ω)	0.9 (decaying linearly to 0.4)
Acceleration coefficients ($c_{1},c_{2}$)	2.0
Number of particles (*P*)	30
Number of iterations (*I*)	100
**Utility Weights and Thresholds**	
Sensor Data Weights ($\omega_{1}\ldots \omega_{5}$)	[0.5, 0.2, 0.1, 0.1, 0.1] (Delay dominant)
Video Service Weights ($\omega_{1}\ldots \omega_{5}$)	[0.1, 0.1, 0.4, 0.3, 0.1] (Bandwidth/Cache dominant)
Assist Threshold ($T_{\mathrm{assist}}$)	0.8 (Activate Helper at 80% load)
Failure Threshold ($T_{\mathrm{fail}}$)	0.95 (Trigger re-election at 95% load)

**Table 2:** Sensitivity Analysis of PSO Hyperparameters and Runtime Performance.

Particles (*P*)	Iterations (*I*)	Avg. Runtime (ms)	Fitness Gain (%)	Remarks
20	50	18.4	−(Baseline)	Under-convergence
**30**	**100**	**42.1**	**+12.0**	**Selected (Optimal)**
50	200	115.6	+14.1	Diminishing Returns

**Table 3:** Comparison of baseline observability and control scope.

Scheme	Observability	Key Metrics Used	Control Scope	Radius	Repl.
Static-RSU	Local	Local association queue	Fixed RSU role; Nearest-RSU association	No	No
AALB [26]	Neighborhood	RSU utilization, Response time, App deadlines	Load balancing via VM/task reassignment	No	No
HRL-PC [19]	Edge–Cloud	Cache states, Content popularity	Proactive cache placement & updates	No	No
PSUV [25]	Global (Path)	Vehicle position/speed, Link quality	PSO-based Routing & Delivery paths	No	No
**PDRA**	**Global (MEC)**	**Entity vector (Load, Context)**	**Role, Radius, & Replication**	**Yes**	**Yes**

## Data Availability

The original contributions presented in this study are included in the article. Further inquiries can be directed to the corresponding author.
